# *rTPI* : An R Package for Calculating Thermal and Aridity Position Indices for Terrestrial Vertebrates

**DOI:** 10.1002/ece3.73397

**Published:** 2026-04-01

**Authors:** Matthew J. Watson, Jeremy T. Kerr

**Affiliations:** ^1^ Department of Biology University of Ottawa Ottawa Ontario Canada

**Keywords:** aridity niche, niche limits, niche position, terrestrial vertebrates, thermal niche

## Abstract

Species environmental niche limits provide useful information for generating estimates of species niche position. Recent work investigating species' responses to climate change has shown that models that included metrics of species‐specific thermal and aridity niche position improve in performance. However, availability to generate thermal and aridity niche limits as well as estimates of niche position has been lacking for large numbers of species. To address the lack of thermal and aridity niche limit data, the *rTPI* package provides access to a dataset of realized thermal and aridity niche tolerance limits for terrestrial birds, mammals, reptiles, and amphibians. Further, it provides users with the ability to generate thermal and aridity niche limit estimates for terrestrial species of interest. Additionally, the package contains functions for calculating Thermal Position Index (TPI) and Aridity Position Index (API) which provide information about species proximity to environmental niche limits. These values can then be utilized in downstream statistical or distribution models aimed at understanding species responses to environmental variables and climate change. *rTPI* provides easy‐to‐use functions and straightforward access to a wide range of realized thermal and aridity niche tolerance limits for a large number of terrestrial vertebrates to be used in addressing a multitude of ecological questions.

## Graphical abstract

Understanding species' niche limits is vital to predicting species‐specific responses to climate change. The rTPI package provides access to a dataset of realized thermal and aridity niche tolerance limits for terrestrial birds, mammals, reptiles, and amphibians. Additionally, the package contains functions for calculating Thermal Position Index (TPI) and Aridity Position Index (API) which provide information about species proximity to environmental niche limits.

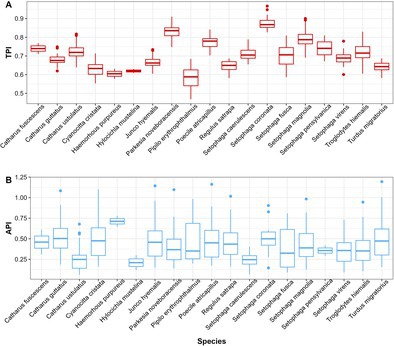

## 1 Introduction

The interactions of species' temperature and aridity tolerance limits are vital determinants of realized niches, when combined with constraints imposed through biotic interactions (Wisz et al. 2013). Species position within their environmental niche can therefore be a useful metric for determining species distributions and vulnerability to environmental change (Bell and Ad Collins 2008; Frishkoff et al. 2015; Thuiller 2004). Due to unique evolutionary histories, thermal and aridity niche limits can vary widely between species (Bozinovic et al. 2014; Calosi et al. 2007; Porter and Kearney 2009). This means that species occupying the same habitat in space and time can display different relative niche positions and would be expected to respond differently to changes in temperature or aridity (Soroye et al. 2020; Williams et al. 2022; Watson and Kerr 2025a, 2025b). This highlights how metrics of species‐specific niche position can have important implications for predicting species' responses to changes in climatic conditions as well as extreme weather events (Germain and Lutz 2020).

Species are anticipated to face increased exposure (frequency and duration) to extreme weather events under climate change (Murali et al. 2023). Models targeted at evaluating species' responses to changing environmental conditions have been found to improve in predictive accuracy when species' physiological tolerances are included (Rezende et al. 2014, Khaliq et al. 2014). However, there is limited availability for data pertaining to species' thermal and aridity physiological tolerance limits (precipitation or aridity limits). This lack of data can make estimating species' position within their environmental niche challenging, sometimes preventing it entirely. One method for addressing the concern of lacking data is through determining species' thermal and aridity niche limits utilizing historical climate data and species range distributions (Kerr et al. 2015). This process provides a method for determining niche limits for large numbers of species that can then be used in the generation of Thermal and Aridity Position Indices (TPI/API) (Kerr 2020). TPI/API provide a scaled metric of species' proximity to their respective environmental niche limits and have been found to improve predictions of species distributions and responses to environmental change (Soroye et al. 2020; Watson and Kerr 2025b; Williams et al. 2022). Further, recent work on bumblebees shows that macroecological‐derived niche limit estimates can be used to generate TPI estimates at microclimatic scales and predict site‐level occupancy (Ednie and Kerr 2022).

Here, we provide a description of the functions and utility of the R package, *rTPI* (v.1.1.1). This package provides access to thermal and aridity niche limits for terrestrial 11,044 bird, 5655 mammal, 9284 reptile, and 7958 amphibian species. Further, this package provides functions generating thermal, aridity, and precipitation niche limit estimates using user supplied species range maps or point observations following the methods in Watson and Kerr (2025a). Additionally, the *rTPI* package provides a suite of functions to calculate Thermal Position Index (TPI) and Aridity Position Index (API) (Equation 1).

(1) $$\mathrm{PI}=\frac{1}{t}\;\sum_{i}^{t}\left(\frac{N_{i}-N_{\min}}{N_{\max}-N_{\min}}\right)$$

PI represents the summed position index (Thermal or Aridity) averaged over a *t* unit (12 if calculated over months of a year). *N*_*i*_ is the environmental measure for a given unit, *N*_min_ and *N*_max_ represent the species‐specific lower and upper niche limits, respectively. For a comprehensive description for the methods used to generate species upper and lower niche limits see Watson and Kerr (2025a). The goal of this package is to provide a direct and efficient method to scale environmental temperature and aridity index (AI) values to species‐specific indices that can be utilized in downstream analyses. We provide an overview of the functions and data contained within the package. Additionally, using an example dataset of species abundance over time, we demonstrate a suggested workflow and ways in which this package can accelerate discoveries related to global change biology and conservation.

## 2 The rTPI Package

The rTPI package (1.1.1) is available from Github (https://github.com/MacroEcoMatt/rTPI) and requires *R* ≥ 3.5.0. Current *rTPI* is hosted on GitHub due to its file size exceeding limitations for packages hosted on CRAN (5 Mb). To install *rTPI* and its dependencies, use the following code: devtools::install_github(‘MacroEcoMatt/rTPI’), or download the .zip file directly from the above URL. *rTPI* contains a set of six functions and three datasets (Table 1). The functions can be divided into three groups based on their utility: verifying genus species names (binomial_check() and syn_check()), extracting data (species_limits() and highertaxa_limits()), and calculating niche limits (api() and tpi()). Datasets containing lower and upper thermal and aridity tolerance limits (Watson and Kerr 2025a) are internally stored within the package to be referenced through functions, but can be extracted to data frames using either the species_limits() or highertaxa_limits() functions. Here, we focus on the intended workflow of functions by group.

**TABLE 1:** List of the main functions and datasets in the *rTPI* package.

Group	Function/dataset name	Brief description
Species Verification	binomial_check()	Compares user submitted species names with dataset species names
Species Verification	syn_check()	Checks user submitted species names with known synonyms
Species Verification	species_list()	Provides a data frame of all species taxonomies within the *rTPI* datasets
Data Extraction	species_limits()	Extracts thermal and aridity limits for provided species
Data Extraction	highertaxa_limits()	Extracts thermal and aridity limits for submitted higher taxonomic groups
Data Extraction	generate_niche()	Allows users to supply their own range maps or species observations to generate niche limit estimates using TerraClimate datasets
Data Extraction	generate_niche_chelsa()	Allows users to supply their own range maps or species observations to generate niche limit estimates using CHESLA monthly datasets
Niche Limit Calculation	api()	Calculates Aridity Position Index
Niche Limit Calculation	tpi()	Calculates Thermal Position Index
Data Extraction/Niche Limit Calculation	month_limits	Contains monthly thermal and aridity limits for terrestrial birds, mammals, reptiles, and amphibians
Data Extraction/Niche Limit Calculation	year_limits	Contains thermal and aridity limits for terrestrial birds, mammals, reptiles, and amphibians averaged over all 12 months
Species Verification	Synonym_list	Contains species binomials used in datasets along with synonymous species names

### 2.1 Species Verification

Due to the presence of synonymous genus species binomials (e.g., *Parus atricapillus* = *Poecile atricapillus* ), we developed two functions that compare user submitted binomials to the binomials used within the niche limits datasets (month_limits and year_limits datasets). We used the taxonomic names provided in the IUCN Red List (2023) as the primary taxonomic source for species genus species binomials within the datasets. Specific taxonomic authorities include Amphibian Species of the World: an Online Reference version 6, Handbook of the Birds of the World and BirdLife International (2023), Mammal Diversity Database v.1.11 (2023), The Reptile Database (Uetz and Hošek 2023). Using the binomial_check() function users can submit a single species binomial or a list of species binomials that will be cross compared with the niche limits datasets. The function returns a data frame with two columns, one containing the user submitted binomial, the second containing a TRUE (binomial matches dataset) or FALSE value (binomial does not match dataset).

If the binomial_match() function returns any FALSE values, the syn_check() function can be used to find the matching binomial in the niche limits datasets with the user submitted binomials. syn_check() accepts a list of species binomials and returns a data frame with a column for submitted binomials and a column containing the matching niche limits dataset binomial. The syn_check() function contains matching accepted taxonomic names and synonyms contained within the Integrated Taxonomic Information System (ITIS 2023), however the function does not account for incorrect spellings. Therefore, if after using syn_check and no matching binomial is found we recommend users consider other packages for identifying misspellings or alternate species synonyms (Chamberlain et al. 2013; Norman et al. 2020). Additionally, users can extract the complete taxonomy for the provided niche limit datasets using the species_list() function.

### 2.2 Data Extraction

Following the verification of species names the species_limits() and highertaxa_limits() functions can be used to extract niche limit data for a list of species binomials or a group of higher level taxa (Class–Genus) respectively. By default, both functions will provide species lower and upper thermal niche limits (°C) and lower and upper aridity niche limits (Aridity Index) for all 12 months. Using species_limits(), the niche limits datasets are filtered and returned using a submitted list of species binomials, variables of interest, and a list of months of interest (can be numeric, e.g., 1, or three letter abbreviation, e.g., Jan). Setting the term yr_avg = TRUE will also include the lower and upper niche limit values averaged over all months. The function highertaxa_limits() provides the same output, but for all members of specified taxonomic groups. Specifying taxonomic level (‘class’ to ‘genus’) and providing a list of names for the taxonomic level will be used to filter the niche limits datasets in this function, rather than specific binomials. Users can then evaluate and utilize the thermal and aridity tolerance limit data at their discretion.

To allow for greater flexibility, we provide two functions (generate_niche(), generate_niche_chelsa()) that allow users to generate niche limit estimates by providing their own species range distributions or point observation datasets. The generate_niche() function uses temperature, precipitation, and potential evapotranspiration data extracted from TerraClimate (Abatzoglou et al. 2018) using the *climateR* (v.0.3.2) package (Johnson 2023) to generate niche limit estimates for any time period between 1961 and 2025 (default 1961–1975). The generate_niche_chelsa performs the same function; however, it uses the *Rchelsa* (v.1.0.2) package (Karger 2025) to extract monthly CHELSA climate data at 1 km resolution, compared to the TerraClimate resolution of 4 km. However, the limitation of the CHELSA dataset is that the earliest year available is 1980 and cannot be used to generate niche limits for the same time period as those provided in the package data (1961–1975). The niche limits generated using these functions can then be used to generate niche position indices by the user (Equation 1).

An important step before calculating TPI and API is ensuring that there are temperature and aridity data for submitted species observations. Temperature data is default calculated using degrees Celsius; however, users can specify either Fahrenheit or Kelvin using the tmp_unit argument to account for different temperature units. Aridity Index values can be calculated by dividing total monthly precipitation by monthly potential evapotranspiration. One important consideration is that the Aridity Index calculation can be prone to extreme values when potential evapotranspiration values are extremely low or precipitation is extremely high relative to potential evapotranspiration (Zomer et al. 2008).

### 2.3 Niche Position Calculation

Once species binomials are matched to the niche limits datasets, the api() and tpi() functions can be used to calculate Aridity Position (API) and Thermal Position Index (TPI) respectively. Data frames submitted to the api() function will be returned with a column listing calculated values for API for that species based on aridity observed at the locations and times the species has been observed. Parameters can be set to include columns of TRUE and FALSE values to quickly filter function output for submitted entries that have incorrect species binomials, incorrectly entered month, or impossible/missing Aridity Index values. Similarly, the functions for tpi() work the same as the api() function, the only difference being the environmental parameter that is calculated. Typically, API and TPI values will fall between 0 and 1, which are the values representing lower (cold/dry) and upper (hot/wet) niche limits. Values that fall below 0 or above 1 are possible and indicate that temperature or aridity conditions at that time exceed a species realized niche limit. Previous studies have found that conditions that approach and exceed the lower/upper bounds of TPI and API are associated with elevated risks of local population extinction (Soroye et al. 2020), population reduction (Williams et al. 2022), and morphological changes (Watson and Kerr 2025b). Kerr (2020) provides a detailed description of TPI.

## 3 A Case Study: Response of Population Abundance to Thermal and Aridity Index Position

In the following example, we use *rTPI* to analyze effects of TPI and API on abundances for several bird species (Dornelas et al. 2018; Hall 1984). After downloading species observation data, we corrected month and day of observation based on the original publication and loaded the data into the R environment. Then, we extracted a list of species binomials from the dataset and compared the list against the niche limit datasets. Of the 20 submitted species binomials, 19 matched the niche limits dataset (Table S1). The binomial that did not match was identified and removed since it only contained the genus name and could not be matched using syn_check() function.

**FIGURE 1:**
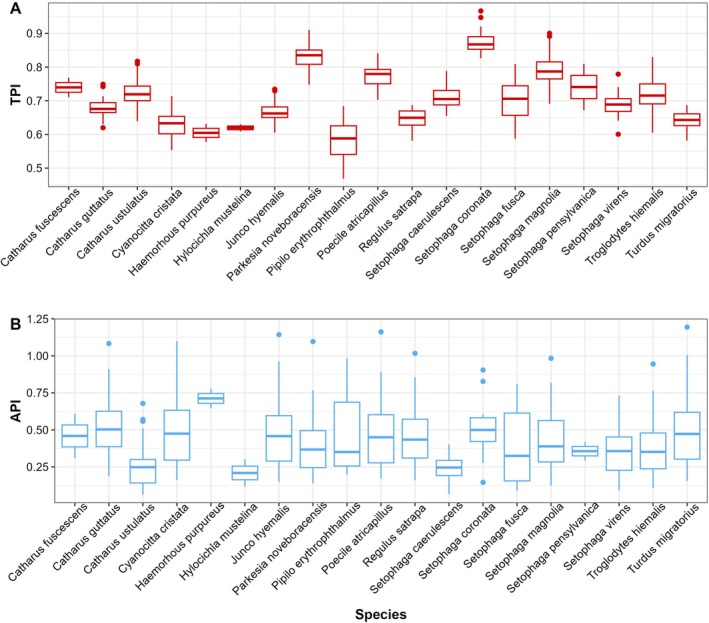
Distribution of niche position values for all species. (A) displays all monthly TPI values. (B) displays all monthly API values.

The bird observation dataset (Hall 1984) had no associated temperature or aridity data included. Using the coordinates provided within the dataset we extracted monthly maximum temperature and Aridity Index from the TerraClimate dataset (Abatzoglou et al. 2018) corresponding to the date and geographic location of each observation. The temperature and aridity values that were extracted for the corresponding months of observation can be passed to the api() and tpi() functions. Since we only have one monthly measurement for AI and temperature, we will leave the ar_var and tmp_var terms as NULL. After completing the api() and tpi() calculations, we merge the two data frames and generate plots to view API and TPI values (Figure 1).

All TPI values and most API values fall within the range of 0 to 1 (Figure 1). Quick evaluation of the plots also shows that species within the dataset exhibit differences in TPI and API values. In other words, within the same habitat, different species maintain populations that differ markedly relative to their upper and lower realized niche limits. At this point, a variety of questions could be investigated using the TPI and API variables. In the original publication, there is no relationship found between temperature and species abundance (Hall 1984). Here, we provide an example re‐analysis of the dataset using species‐specific values for TPI and API.

First, we averaged Abundance, TPI, and API values within each year of observation for all species and then removed all species with only a single observation (*n* = 5). We then generated a linear mixed effect model following a Bayesian framework to evaluate the response of population abundance to TPI and API values over time. Population abundance served as our response variable, while TPI, API, and year of observation served as our predictor variables. All model variables were z‐transformed prior to analysis due to differences in scale between TPI/API and year and to better allow for the comparison of coefficient magnitudes between TPI and API. We accounted for temporal autocorrelation between population abundances and year by including a first order autoregressive process, grouped by species identity. We additionally included species identity as a random intercept, allowed for random slopes for all predictor variables across each species. We included weakly informative priors in our model, normal priors for the intercept and slope terms (mean = 0, SD = 5), and a Half‐Cauchy prior for the random effect terms (median = 0, scale = 5). We ran our model with a warmup phase of 500 iterations followed by a sampling phase of 1000 iterations over 4 chains. All chains were found to converge (Figures S1–S3), and a posterior predictive check showed good model fit (Figure S4). To compare results generated using TPI and API with raw weather data, we generated a second model replacing TPI with mean monthly max temperature () and mean monthly Aridity Index (AI) and keeping all other model details identical and followed the same model inspection procedure (Figures S5–S7).

**FIGURE 2:**
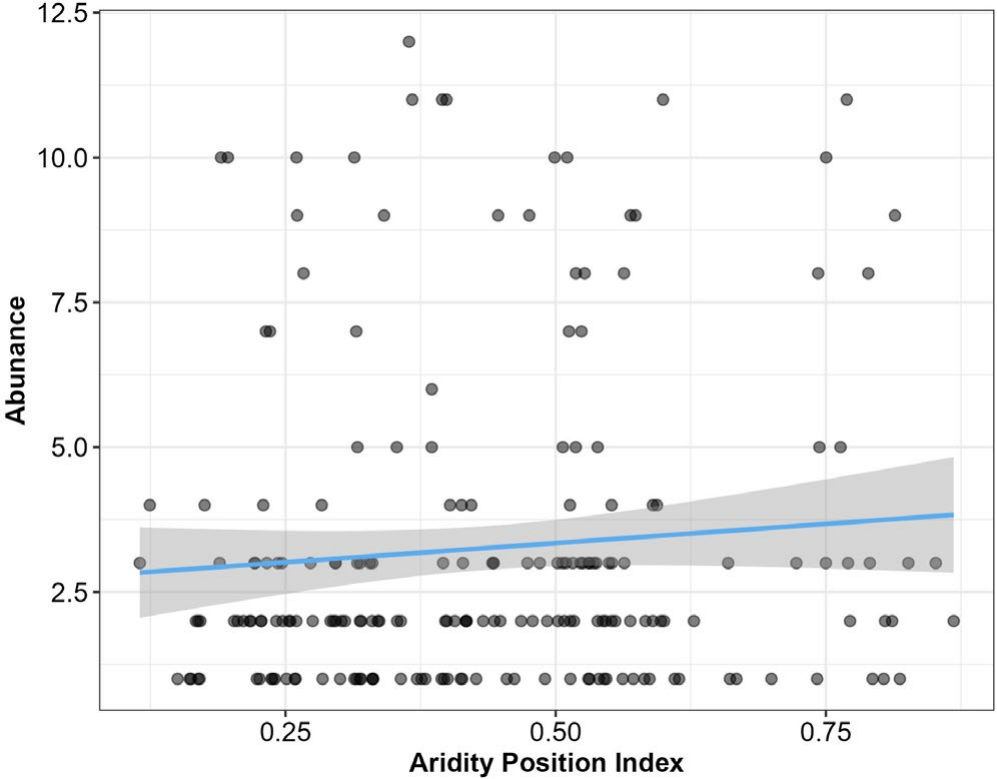
Response of species' abundances in response to Aridity Position Index (API).

As in the original study, temperature, or in this case TPI, did not predict abundance (Est = 0.097, 95% CI = −0.139 to 0.330) (Table 2). However, we did observe that increasing API resulted in increased abundance (Est = 0.100, 95% CI = 0.012–0.186) (Table 2). This indicates that species' abundances increase when species experience conditions that are closer to their upper API limits (wetter limits) (Figure 2). In comparison, when we conducted the same analysis using raw weather variables (temperature and Aridity Index), we observed no significant effect of temperature (Est = 0.025, 95% CI = −0.701 to 0.185) or Aridity Index (Est = 0.081, 95% CI = −0.007 to 0.169) on bird abundance (Table S2). This demonstrates that accounting for species‐specific niche values provides important information when evaluating species responses to environmental variables.

**TABLE 2:** Linear mixed effect model results for the analysis of population abundance response to TPI and API.

Variable	Estimate	SE	95% CI	Bulk ESS
*Regression Coefficients*				
Intercept	−0.026	0.229	−0.717 to 0.177	904
TPI	0.097	0.119	−0.139 to 0.330	3512
**API**	**0.100**	**0.045**	**0.012 to 0.186**	**4055**
Year	0.420	0.123	−0.284 to 0.195	1604
*Multilevel Hyperparameters*				
Intercept	0.849	0.192	0.560 to 1.297	1460
TPI	0.116	0.090	0.006 to 0.336	16,266
API	0.058	0.042	0.003 to 0.156	1808
Year	0.420	0.101	0.267 to 0.658	1695
*Distributional Parameters*				
Sigma	0.366	0.021	0.329 to 0.411	4029

*Note:* Estimates represent the change in mean abundance based on a 1 SD increase from the mean value of the respective variable. Significance is indicated when the 95% Credible Interval (95% CI) does not encompass 0. All variables displayed an *R*‐hat value of 1 indicating sampling convergence. Temporal autocorrelation structures are weak but significant (Est = 0.290, 95% CI = 0.112–0.476). Bulk effective sample size (Bulk ESS) is greater than 400 (100 per chain) indicating efficient sampling quality. Bold values indicate significant estimates based on 95% credible interval not spanning 0 and are no associated *p* values with Bayesian analysis.

Approaching upper API limits is associated with greater water availability in the environment and would be associated with increased primary productivity (Golodets et al. 2015). This increase in productivity could result in greater food availability relative to species requirements, which could drive higher carrying capacity in the environment within a given year and result in increased abundance (Hanses et al. 2011). Alternatively, this result can be interpreted to indicate that abundances decline as species approach their lower API (dry limits). This could be a result of the inverse effect mentioned previously, where drier conditions would result in decreased food abundance. Combined with decreased food abundance, drier conditions (lower API) increase the risk of dehydration through evaporative water loss (Song and Beissinger 2020), which would further impact species survival and abundance. All code, model evaluation, and full results can be found in the Data S1. For more detailed studies on TPI and API impacts see: Soroye et al. (2020); Watson and Kerr (2025a, 2025b); Williams and Newbold (2021).

## 4 Concluding Remarks and Future Directions

Species are facing rapid changes in environmental conditions due to climate change, which exerts profound impacts on phenology, range shifts, and extinction risk (Cahill et al. 2013; Chen et al. 2011; Visser and Both 2005). Understanding how species are predicted to respond has become increasingly vital for conservation planning, which will be aided by the ability to account for differences in environmental niche limits between species (Rezende et al. 2011; Sunday et al. 2012). In fact, models of species responses to environmental change have been shown to improve when species‐specific niche positions are accounted for using physiological tolerances (Cooper et al. 2008; Rezende et al. 2014; Taylor et al. 2021). However, challenges exist for incorporating species‐specific physiological tolerances due to data availability or limited transferability of controlled studies of species' thermal tolerances to natural conditions (Rezende et al. 2014).

Here, we provide a methodology for efficiently converting vital temperature and aridity measurement to species‐specific tolerance indices (TPI and API). TPI has previously been found to accurately predict species range shifts and population response to climate and land use change (Soroye et al. 2020; Williams et al. 2022; Williams and Newbold 2021). However, it is important that we specify that thermal and aridity niche limits detailed here are not meant to be interpreted as physiological tolerances. For a detailed description of the methodologies, applications, and limitations of the thermal and aridity niche limit datasets along with comparisons to physiological tolerances see Watson and Kerr (2025a). The breadth of taxonomic coverage of the TPI and API data along with the support of previous studies makes this an effective method to account for species‐specific tolerances in future studies. Potential studies that could benefit from the incorporation of TPI and API could include those that evaluate how environmental factors impact population trends, phenological shifts, and range shifts. Additionally, conservation planning would benefit from utilizing TPI and API for predicting changes to community composition, future species distributions, and species persistence potential. We hope that the *rTPI* package provides an effective tool to enable future researchers to incorporate detailed species‐specific thermal and aridity tolerances into their research design and analyses.

## Author Contributions

**Matthew J. Watson:** conceptualization (equal), data curation (lead), formal analysis (lead), investigation (lead), methodology (equal), software (lead), validation (lead), visualization (lead), writing – original draft (lead), writing – review and editing (equal). **Jeremy T. Kerr:** conceptualization (equal), data curation (supporting), formal analysis (supporting), funding acquisition (lead), methodology (equal), project administration (lead), resources (lead), supervision (lead), writing – original draft (supporting), writing – review and editing (equal).

## Funding

This work was supported by the Natural Sciences and Engineering Research Council of Canada.

## Conflicts of Interest

The authors declare no conflicts of interest.

## Supporting information


**Data S1:** ece373397‐sup‐0001‐Supinfo1.csv.

**Figure S1:** Density and trace plots for all model variables showing normal distribution of model estimates, and complete chain convergence for the model using TPI and API (Table 2).
**Figure S2:** Density distribution and trace plots showing complete chain convergence for random effect term estimates for the model using TPI and API (Table 2).
**Figure S3:** Density distribution and trace plots showing complete chain convergence for distribution (sigma) and autocorrelation parameters for the model using TPI and API (Table 2).
**Figure S4:** Posterior Predictive Check plot of linear mixed effect model using TPI and API (Table 2).
**Figure S5:** Density and trace plots for all model variables showing normal distribution of model estimates, and complete chain convergence for the model using raw weather variables (Table S2).
**Figure S6:** Density distribution and trace plots showing complete chain convergence for random effect term estimates and autocorrelation parameters for the model using raw weather variables (Table S2).
**Figure S7:** Posterior Predictive Check plot of linear mixed effect model using raw weather variables (Table S2).
**Table S1:** List of species with common name included in our analysis with associated thermal and aridity niche limit data.
**Table S2:** Model results for analysis of abundance response to raw weather variables. All variables were *z*‐transformed prior to analysis to aid in comparison of the strength of model coefficients. 95% CI represents 95% credible intervals, with 95% CI not overlapping 0 representing significance. AR1 correlation structure = 0.292 (SE = 0.093), distributional parameter (sigma) = 0.369 (SE = 0.021).

## Data Availability

rTPI is available at Github (https://github.com/MacroEcoMatt/rTPI). Data used for the demonstration of *rTPI* functions was accessed from BioTIME (https://doi.org/10.1111/geb.12729, date accessed: Apr 09, 2025) and the original dataset was collected by the Brooks Bird Club and published by George A. Hall in 1984 (https://www.jstor.org/stable/4161914).
