# Supplementary material for: rTPI : An R Package for Calculating Thermal and Aridity Position Indices for Terrestrial Vertebrates
Source: Ecol Evol. 2026 Apr 1;16(4):e73397. doi: 10.1002/ece3.73397 (PMC13045246; doi:10.1002/ece3.73397)
Supplement: Supplementary file 2 — Figure S1: Density and trace plots for all model variables showing normal distribution of model estimates, and complete chain convergence for the model using TPI and API (Table 2). Figure S2: Density distribution and trace plots showing complete chain convergence for random effect term estimates for the model using TPI and API (Table 2). Figure S3: Density distribution and trace plots showing complete chain convergence for distribution (sigma) and autocorrelation parameters for the model using TPI and API (Table 2). Figure S4: Posterior Predictive Check plot of linear mixed effect model using TPI and API (Table 2). Figure S5: Density and trace plots for all model variables showing normal distribution of model estimates, and complete chain convergence for the model using raw weather variables (Table S2). Figure S6: Density distribution and trace plots showing complete chain convergence for random effect term estimates and autocorrelation parameters for the model using raw weather variables (Table S2). Figure S7: Posterior Predictive Check plot of linear mixed effect model using raw weather variables (Table S2). Table S1: List of species with common name included in our analysis with associated thermal and aridity niche limit data. Table S2: Model results for analysis of abundance response to raw weather variables. All variables were z‐transformed prior to analysis to aid in comparison of the strength of model coefficients. 95% CI represents 95% credible intervals, with 95% CI not overlapping 0 representing significance. AR1 correlation structure = 0.292 (SE = 0.093), distributional parameter (sigma) = 0.369 (SE = 0.021). [file ECE3-16-e73397-s001.docx]

# Supplementary Materials

# rTPI: An R package for calculating Thermal and Aridity Position Indices for terrestrial vertebrates

# Workflow Code:

#setwd()

#devtools::install_github("MacroEcoMatt/rTPI")
library(rTPI)
library(dplyr)

##
## Attaching package: 'dplyr'

## The following objects are masked from 'package:stats':
##
## filter, lag

## The following objects are masked from 'package:base':
##
## intersect, setdiff, setequal, union

bird_data <- read.csv("Bird_Data.csv")

bin_list <- unique(bird_data$Binomial)
check_list <- binomial_check(bin_list)

bird_data <- bird_data %>% filter(!Binomial=="Vireo")

bird_data_tpi <- tpi(bird_data, tmp_var = NULL)

bird_data <- bird_data %>% rename(Ar = aridity)

bird_data_api <- api(bird_data, ar_var = NULL)

Final_data <- left_join(bird_data_tpi,bird_data_api)

## Joining with `by = join_by(X, ABUNDANCE, Binomial, Lat, Lon, Day, Month, Year,
## Tm, pet, ppt, Ar, Date)`

f_data <- Final_data %>% group_by(Binomial,Year) %>%
 summarise(ABUNDANCE = mean(ABUNDANCE),
 TPI = mean(TPI),
 API = mean(API), Ar = mean(Ar), tmax = mean(tmax)) %>%
 group_by(Binomial) %>% filter(n() > 1)

## `summarise()` has grouped output by 'Binomial'. You can override using the
## `.groups` argument.

mfinal <- brm(scale(ABUNDANCE) ~ scale(TPI) + scale(API) + scale(Year)

+ ar(time = Year, gr = Binomial, p = 1, cov = FALSE)

+ (1 + scale(TPI) + scale(API) + scale(Year)||Binomial),

data = df_final, family=gaussian(),

prior = c(set_prior("normal(0,5)", class = "Intercept"),

set_prior("normal(0,5)", class = "b"),

set_prior("cauchy(0,5)", class = "sd")),

warmup = 500, iter = 1500, chains = 4, cores = 4,

backend = "cmdstanr", normalize = F,

control = list(adapt_delta = 0.9))

plot (mfinal)

pp_check(mfinal)

mfinal_raw <- brm(scale(ABUNDANCE) ~ scale(tmax) + scale(Ar) + scale(Year)

+ ar(time = Year, gr = Binomial, p = 1, cov = FALSE)

+ (1 + scale(tmax) + scale(Ar) + scale(Year)||Binomial),

data = df_final, family=gaussian(),

prior = c(set_prior("normal(0,5)", class = "Intercept"),

set_prior("normal(0,5)", class = "b"),

set_prior("cauchy(0,5)", class = "sd")),

warmup = 500, iter = 1500, chains = 4, cores = 4,

backend = "cmdstanr", normalize = F,

control = list(adapt_delta = 0.9))

plot (mfinal)

pp_check(mfinal)

plot (mfinal_raw)

pp_check(mfinal_raw)

library(ggthemes)

library(ggeffects)

library(ggplot2)

library(sjPlot)

theme_set(theme_sjplot())

api_mod <- ggplot(f_data, aes(x=API, y = ABUNDANCE))+

geom_point(alpha=0.5,size=2)+

geom_smooth(method="lm", linewidth=1, color="steelblue2")+

xlab("Aridity Position Index")+ ylab("Abunance")+

theme_bw()+

theme(axis.title = element_text(face="bold",size=12, color="black"),

axis.text = element_text(size=12, color="black"),

legend.text = element_text(color="black",size=10, face="bold"))

**
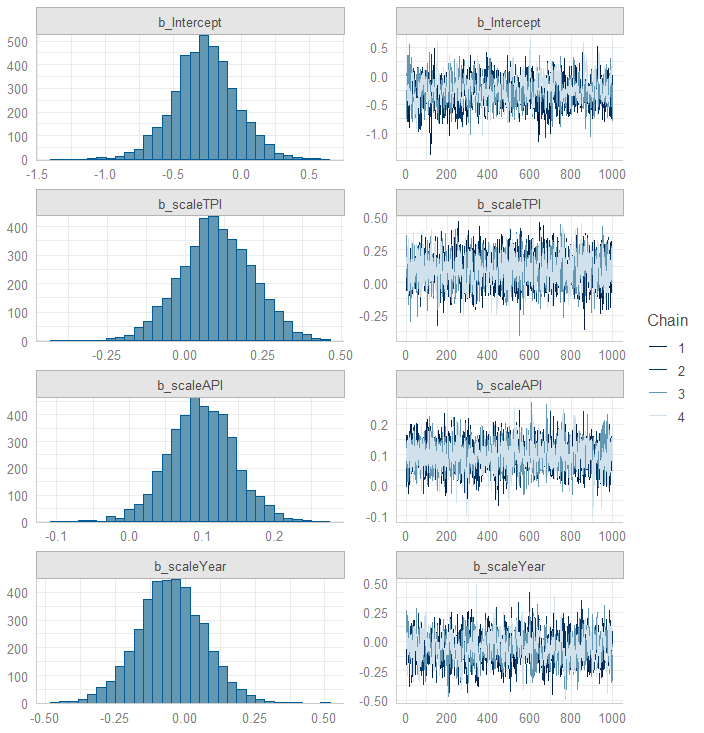
**

**Figure S1.** Density and trace plots for all model variables showing normal distribution of model estimates, and complete chain convergence for the model using TPI and API (Table 2).

**
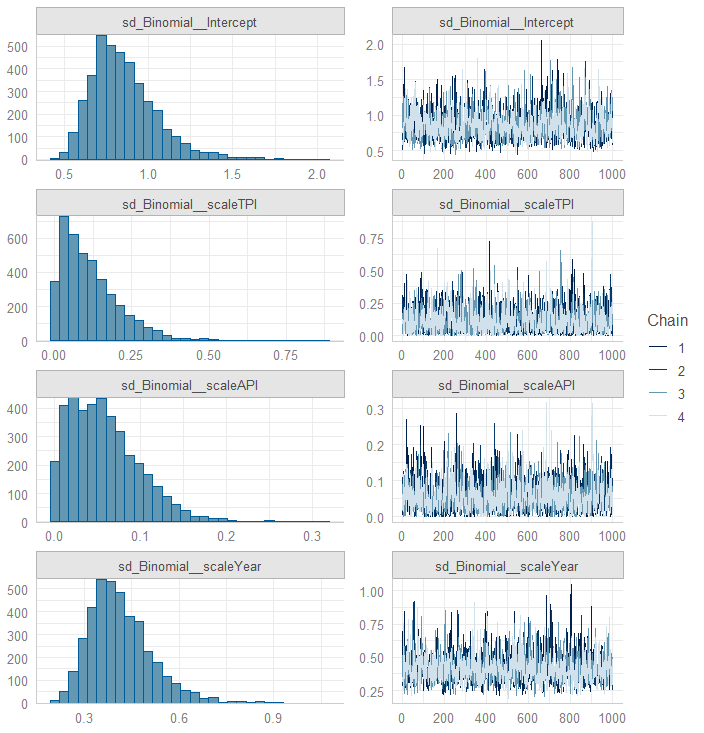
**

**Figure S2.** Density distribution and trace plots showing complete chain convergence for random effect term estimates for the model using TPI and API (Table 2).

**
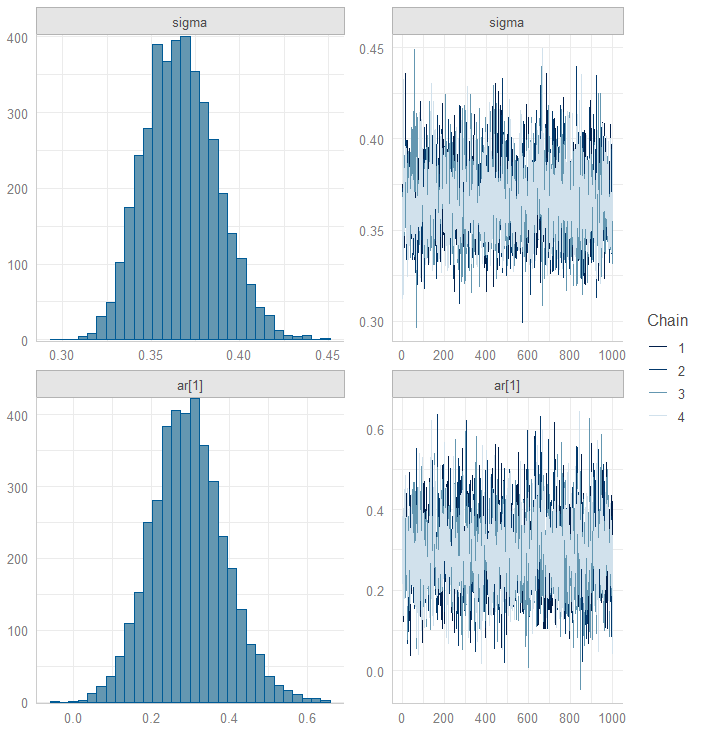
**

**Figure S3.** Density distribution and trace plots showing complete chain convergence for distribution (sigma) and autocorrelation parameters for the model using TPI and API (Table 2).

**
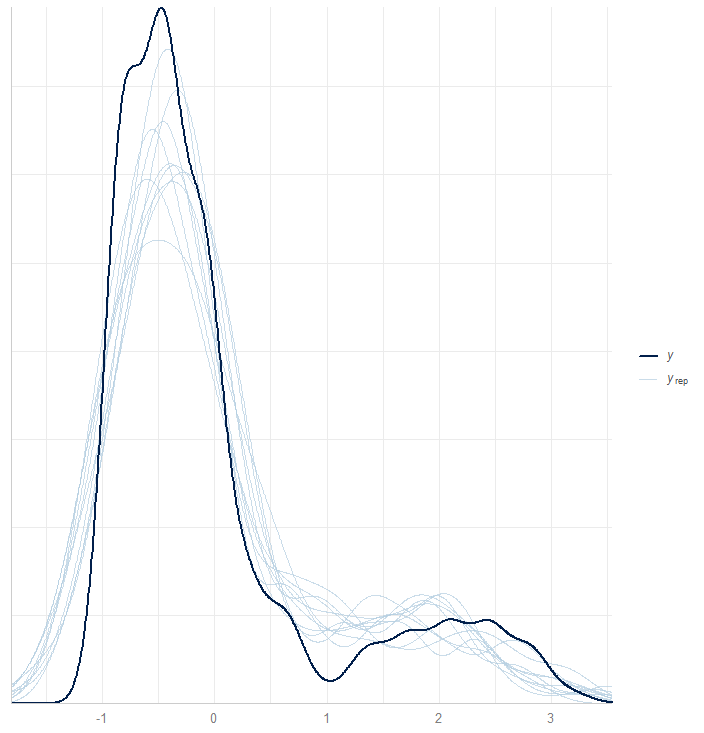
**

**Figure S4.** Posterior Predictive Check plot of linear mixed effect model using TPI and API (Table 2).

**
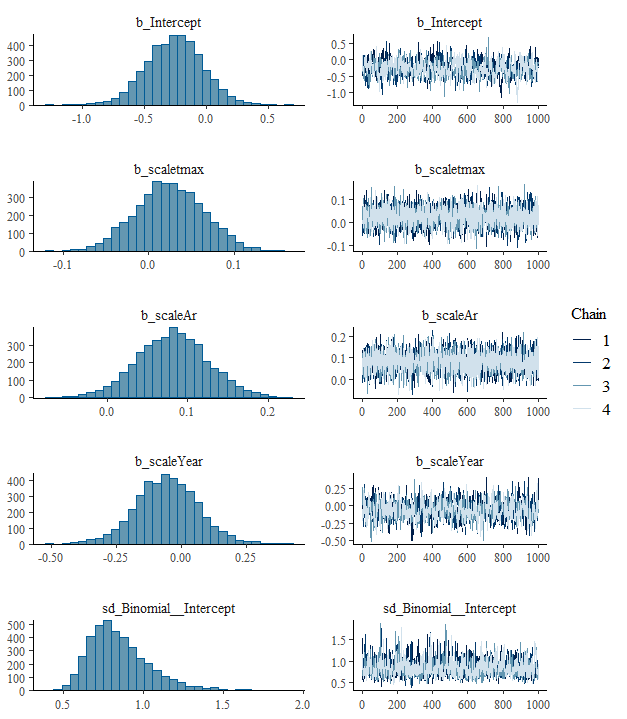
**

**Figure S5.** Density and trace plots for all model variables showing normal distribution of model estimates, and complete chain convergence for the model using raw weather variables (Table S2).

**
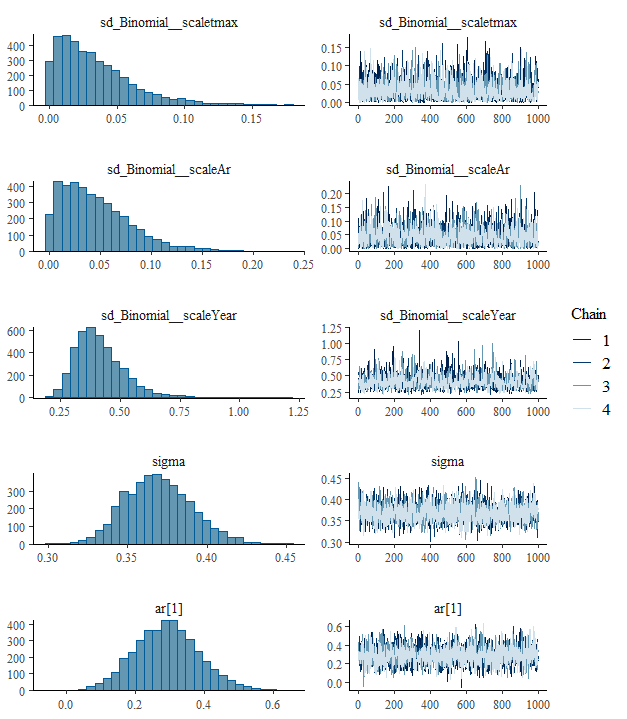
**

**Figure S6.** Density distribution and trace plots showing complete chain convergence for random effect term estimates and autocorrelation parameters for the model using raw weather variables (Table S2).


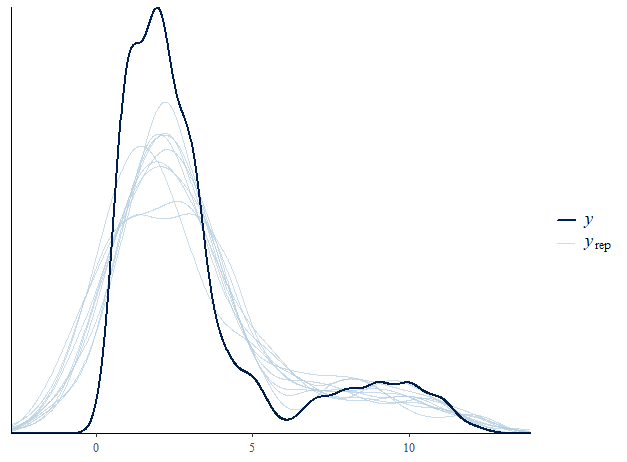


**Figure S7.** Posterior Predictive Check plot of linear mixed effect model using raw weather variables (Table S2).

**Table S1. List of species with common name included in our analysis with associated thermal and aridity niche limit data.**

| **Species Binomial (*Genus species)*** | **Common Name** |
| --- | --- |
| *Catharus fuscescens* | Veery |
| *Catharus guttatus* | Hermit thrush |
| *Catharus ustulatus* | Swainson's thrush |
| *Cyanocitta cristata* | Blue jay |
| *Haemorhous purpureus* | Purple finch |
| *Hylocichla mustelina* | Wood thrush |
| *Junco hyemalis* | Dark-eyed junco |
| *Parkesia noveboracensis* | Northern waterthrush |
| *Pipilo erythrophthalmus* | Eastern towhee |
| *Poecile atricapillus* | Black-capped chickadee |
| *Regulus satrapa* | Golden-crowned kinglet |
| *Setophaga caerulescens* | Black-throated blue warbler |
| *Setophaga coronata* | Yellow-rumped warbler |
| *Setophaga fusca* | Blackburnian warbler |
| *Setophaga magnolia* | Magnolia warbler |
| *Setophaga pensylvanica* | Chestnut-sided warbler |
| *Setophaga virens* | Black-throated green warbler |
| *Troglodytes hiemalis* | Winter wren |
| *Turdus migratorius* | American robin |

**Table S2. Model results for analysis of abundance response to raw weather variables.** All variables were z-transformed prior to analysis to aid in comparison of the strength of model coefficients. 95% CI represents 95% credible intervals, with 95% CI not overlapping 0 representing significance. AR1 correlation structure = 0.292 (SE = 0.093), distributional parameter (sigma) = 0.369 (SE = 0.021).

| **Fixed Effects** | | | | |
| --- | --- | --- | --- | --- |
| *Variable* | *Estimate* | *SE* | *95% CI* | *R-hat* |
| Intercept | -0.257 | 0.228 | -0.701 - 0.185 | 1.015 |
| Tmax | 0.025 | 0.040 | -0.053 - 0.103 | 1.000 |
| AI | 0.081 | 0.044 | -0.007 - 0.169 | 1.000 |
| Year | -0.057 | 0.122 | -0.302 - 0.184 | 1.004 |
| **Random Effects (~Binomial)** | | | | |
| *Variable* | *Estimate* | *SE* | *95% CI* | *R-hat* |
| Intercept | 0.847 | 0.187 | 0.570 - 1.282 | 1.003 |
| Tmax | 0.033 | 0.027 | 0.001 - 0.101 | 1.003 |
| AI | 0.046 | 0.036 | 0.002 - 0.131 | 1.002 |
| Year | 0.415 |  | 0.262 - 0.659 | 1.001 |
